# Genome-Wide Association and Expression Analysis of the Lipoxygenase Gene Family in *Passiflora edulis* Revealing *PeLOX4* Might Be Involved in Fruit Ripeness and Ester Formation

**DOI:** 10.3390/ijms232012496

**Published:** 2022-10-18

**Authors:** Dongmei Huang, Funing Ma, Bin Wu, Wenhui Lv, Yi Xu, Wenting Xing, Di Chen, Bingqiang Xu, Shun Song

**Affiliations:** 1 Key Laboratory of Genetic Improvement of Bananas, Haikou Experimental Station/Hainan Key Laboratory for Biosafety Monitoring and Molecular Breeding in Off-Season Reproduction Regions, Sanya Research Institute of Chinese Academy of Tropical Agricultural Sciences, Chinese Academy of Tropical Agricultural Sciences, Haikou 571101, China; 2 Hainan Yazhou Bay Seed Laboratory, Sanya 572000, China; 3 School of Tropical Crops, Yunnan Agricultural University, Puer 665000, China

**Keywords:** lipoxygenase (LOX), gene family, passion fruit, expression analysis, volatile ester

## Abstract

Aroma is an important factor in fruit quality. *Passiflora edulis* (passion fruit) is popular among consumers because of its rich flavor and nutritional value. Esters are the main components of the volatile aroma of passion fruit. Lipoxygenase (LOX), as the first key enzyme upstream of esters, may play an important role in the formation of passion fruit aroma. In this study, a total of 12 passion fruit LOX (*PeLOX*) members were screened out based on the *Passiflora edulis* genome database, which were distributed unevenly on 6 chromosomes, all containing the highly conserved lipoxygenase domain and some containing the PLAT domain. The gene structure, evolutionary analysis and cis-acting elements of the family members were predicted in this study. Transcriptome analysis showed that 12 *PeLOX* genes had different degrees of response to different abiotic stresses (drought stress, salt stress, cold stress, and high temperature). *PeLOX1*, *PeLOX2*, *PeLOX7*, *PeLOX11*, and *PeLOX12* responded significantly to various abiotic stresses, while *PeLOX8* and *PeLOX9* had little change in expression in all stresses. Quantitative real-time PCR (qRT-PCR) in six tissues revealed that the 12 *PeLOX* genes exhibited tissue expression specificity, and the relative expression of most genes were particularly high in the roots, stems, and fruits. Focusing on passion fruit ripening and ester synthesis, the transcriptomic analysis showed that with the increase in fruit development and fruit maturity, the expression levels of *PeLOX1*, *PeLOX9*, *PeLOX11*, and *PeLOX12* showed downregulated expression, while *PeLOX2* and *PeLOX4* showed upregulated expression. In particular, the upregulation trend of *PeLOX4* was the most obvious, and the qRT-PCR results were consistent with the transcriptome result. Pearson correlation analysis showed that with the development and ripening of fruit, the expression level of *PeLOX4*, LOX enzyme activity and total ester content all showed an increasing trend, in particular during the period when the peel was red and shrank (from T2 to T3 stage), the esters’ contents increased by 37.4 times; the highest expression levels were all in the T3 period. The results indicated that *PeLOX4* may be a candidate gene involved in fruit ripeness and the formation of volatile aroma compounds, with the increase in fruit ripening, the expression level of *PeLOX4* increased and the LOX enzyme activity increased accordingly, thereby promoting the synthesis of volatile esters in fruit pulp. Our discovery lays the foundation for the functional study of LOX in passion fruit.

## 1. Introduction

*Passiflora edulis* Sims is a perennial evergreen vine of the *Passiflora* genus, which is also known as passion fruit [1]. It can emit the rich fragrance of dozens of fruits, such as banana, pineapple, guava, lemon, strawberry, mango, and other fruits. Due to its rich aroma and nutrients, passion fruit is known as the “king of juices” and “king of beverages” [2]. Aroma is one of the most important quality traits of passion fruit. It is of great significance to explore the candidate genes related to its synthesis and their functions.

The volatile aroma of passion fruit mainly includes esters, alcohols, aldehydes, ketones and terpenes. In our previous study, a total of 142 aromatic compounds were detected, among which esters accounted for 80.31% at the maturity stage and were the main compounds contributing to the aroma of passion fruit pulp [2]. Other reports also supported the notion that esters were the main aroma compounds of passion fruit [3,4]. Ester volatiles are synthesized by metabolic precursors of fatty acids and specific amino acids under the regulation of multiple enzymes [5]. The fatty acid metabolism LOX pathway is the key source of aromatic volatile ester precursors in many fruits [6]. Lipoxygenase (LOX, EC 1.13.11.12) is the key enzyme in the first step of this pathway; it is an enzyme that does not contain heme iron and can initiate the degradation of polyunsaturated fatty acids (PUFA) into hydroperoxides [7]. According to the different oxygenation sites catalyzed by LOX, it is divided into 9-LOX and 13-LOX. These intermediate hydroperoxides can generate aroma compounds such as aldehydes, alcohols, and esters through the hydroperoxide lyase (HPL) pathway under the action of alcohol dehydrogenase (ADH) and alcohol acyltransferase (AAT), and can also produce jasmonates (jasmonic acid and methyl jasmonate) via the allene oxide synthase (AOS) pathway. The jasmonate synthetic pathway plays a role in resistance to stress and is associated with plant growth, development, stress resistance, maturation, and senescence [8]. The inducibility of LOX in the leaves of passion fruit in response to herbivory [9], insect [10] and pathogen [11] attack, and the application of jasmonate [12] has been extensively studied. However, there is no relevant report on its relationship with the formation of flavor substances.

The LOX family is widely distributed in plants and encoded by multiple gene families. In recent years, increasing numbers of members of the LOX gene family have been discovered or analyzed, and 6, 18, 12, 23, 25, 18, 23, 12, and 14 family members were identified in Arabidopsis [13], grape [14], rice [15], cucumber [16], apple [7], melon [17], pear [18], peach [19], and tomato [20], respectively. Some LOX genes have been found to play a role in various physiological processes, such as fruit development, ripening, and volatile substance formation. Apple ripening is accompanied by an increase in the autonomous emissions of hexanol and esters derived from hexanol (hexyl esters), and these compounds are dependent upon hexanal synthesis by the action of lipoxygenase (LOX) on fatty lipids [21]; meanwhile, *MdLOX1a* and *MdLOX5e* were identified as candidate genes to be involved in fruit aroma volatile production [7]. The transcription levels of *PpLOX1* and *PpLOX4* in peach are upregulated with the increase in ethylene accumulation during fruit ripening, and are involved in the synthesis of volatile aroma compounds [22]. In melon, *CmLOX18* may play an important role in the synthesis of C6 compounds [23]. The expression of *PuLOX3* and other genes in “Nanguo Pear” contributed the most to the changes in total esters and major esters in fruit aroma [24].

At present, there are few studies on the functional gene-mining of passion fruit and its formation mechanism, especially in terms of growth and development, fruit ripening, and stress resistance. In the previous work [2], an important metabolic pathway for the aroma synthesis of passion fruit pulp and the *PeLOX* gene in the formation of esters were obtained. In this study, high-quality passion fruit genome-wide data were used to analyze the information on *PeLOX* family members and obtain the specific expression of their family members in different tissues. At the same time, the role of *PeLOX* genes in abiotic stress resistance and fruit ripening were analyzed, and the key gene *PeLOX4*′s expression level, enzyme activity, and ester content in ester synthesis were mainly detected; moreover, the expression trend of the detection indicators was analyzed. These works provide a theoretical basis for the mining of key functional genes and the genetic improvement of passion fruit.

## 2. Results and Analysis

### 2.1. Identification of LOX Gene Family Members

**Figure 1:**
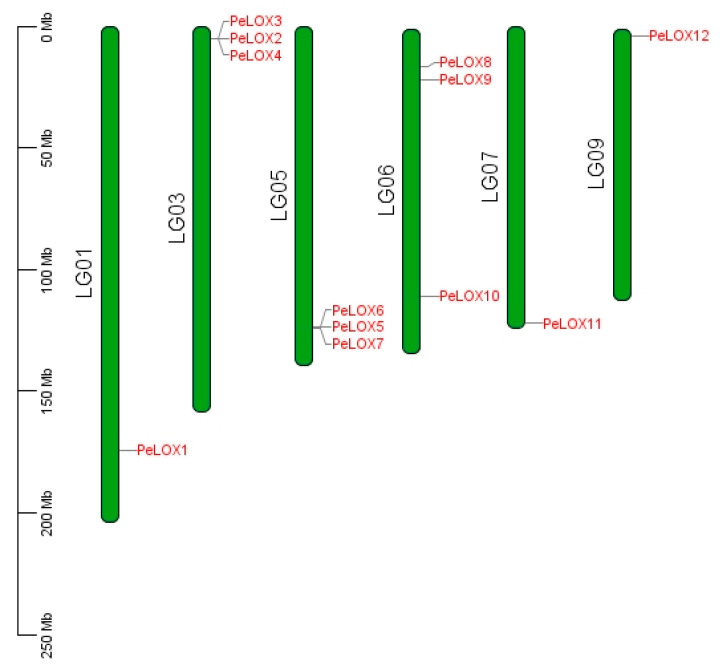
Distribution of *PeLOX* family on *Passiflora edulis* chromosomes.

A total of 18 possible LOX members were retrieved by BlastP and HMM Search. Six short sequences and incomplete sequences of the lipoxygenase domain were removed after SMART filtering, and, finally, 12 *PeLOX* protein sequences were obtained (Table 1). In total, 12 *PeLOX* genes were distributed on 6 chromosomes (Figure 1); *PeLOX2/3/4* and *PeLOX5/6/7* were connected in tandem and distributed on chromosomes 3 and 5, respectively, indicating their high homology and possibly similar functions. The amino acid sequence length ranged from 472 to 933 aa, with an average length of 822 aa, and the theoretical pI ranged from 5.42 to 9.16. Subcellular location prediction results showed that all 12 *PeLOX* proteins were located in the cytoplasm, nucleus, and chloroplast. The secondary structure prediction results showed that the α-helix (30.61–46.40%), extended chain (1.69–8.39%), and random coils (51.89–64.32%) were scattered throughout the protein, with random coils accounting for the majority.

In addition, 6 LOX proteins were obtained from Arabidopsis, 23 from apple, and 15 from tomato, with a total of 56 proteins. The sequence location information and characteristic information are shown in Table S1 (Supplementary Materials).

### 2.2. Phylogenetic Analysis

**Figure 2:**
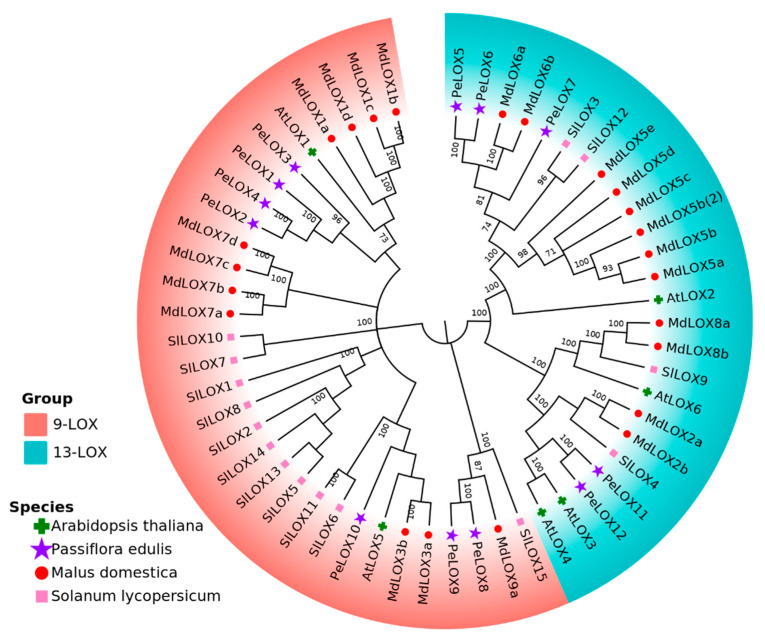
Phylogenetic tree for the protein for the LOX gene family in passion fruit, *Arabidopsis thaliana*, apples, and tomato.

The LOX proteins of passion fruit, Arabidopsis thaliana, apple, and tomato were analyzed by MAFFT, and the phylogenetic tree was constructed using the MEGA-X software via the neighbor-joining (N-J) method (Figure 2). Cluster analysis showed that the 56 proteins could be divided into two groups: Group 1, which was 9-LOX, including *PeLOX1*, *PeLOX2*, *PeLOX3*, *PeLOX4*, *PeLOX8*, *PeLOX9*, and *PeLOX10*; and Group 2, which was 13-LOX, including *PeLOX5*, *PeLOX6*, *PeLOX7*, *PeLOX11*, and *PeLOX12*. Sequences located in different branches of the same group in the evolutionary tree have a high similarity in gene structure, indicating that the LOX sequences of the four species had high homology and conservation.

### 2.3. Analysis of PeLOX Gene Family Structure

**Figure 3:**
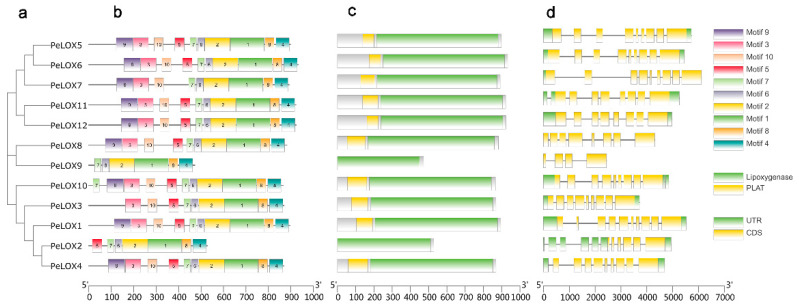
Phylogenetic relationships, gene architecture of conserved protein motifs and gene structure in *PeLOX* genes. (**a**) Phylogenetic tree based on the *PeLOX* genes sequence. (**b**) The motif composition of *PeLOX* family. Different colored boxes display different motifs. (**c**) Batch CD result of *PeLOX* genes, most of the members contained the conserved Lipoxygenase and PLAT domains. (**d**) The exon–intron structure of *PeLOX* genes.

**Figure 4:**
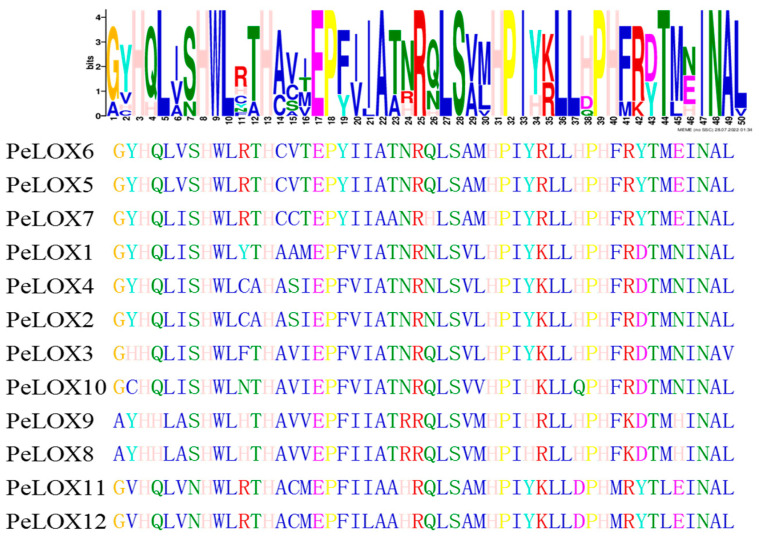
Sequence alignments of a conservative motif His-(X)_4_-His-(X)_4_-His-(X)_17_-His-(X)_8_-His in LOX proteins of passion fruit.

TBtools was used to visualize the phylogenetic relationships, the gene architecture of conserved protein motifs, and the gene structure in the *PeLOX* family (Figure 3). Proteins with high homology had similar recognition motifs. Most members had 9–10 similar motifs, while *PeLOX9* had 6 motifs, and *PeLOX2* had 7 motifs. In the conservative domain structure search results, *PeLOX2* and *PeLOX9* lacked the PLAT domain. Therefore, the motifs missing in these two proteins might be the sequences constituting the PLAT domain. Among the 12 *PeLOX* motifs, there was a conserved sequence, His-(X)_4_-His-(X)_4_-His-(X)_17_-His-(X)_8_-His (Figure 4). LOX was a protein containing non-heme iron.

Exon–intron structure is an important feature of gene evolution. We further analyzed the exon–intron structure of *PeLOX* genes. Most members contained the 5′UTR (untranslated region) and 3′UTR, except for *PeLOX8* and *PeLOX9*, which had no UTR structure. In addition, *PeLOX7* only had a 5′UTR structure and no 3′UTR, and *PeLOX2* contained multiple 5′UTRs. Most family members contained 8–9 exons, while *PeLOX1* had 10 exons, and *PeLOX9* and *PeLOX2* had only 4 and 5 exons, respectively. In general, with the exception of the shorter sequences of *PeLOX2* and *PeLOX9*, combining the results of phylogenetic relationships, the gene architecture of conserved protein motifs, and gene structure analysis, the gene architecture of conserved protein motifs and sequence homology of the 12 *PeLOX* genes are highly similar. 

### 2.4. Cis-Acting Element Prediction

**Figure 5:**
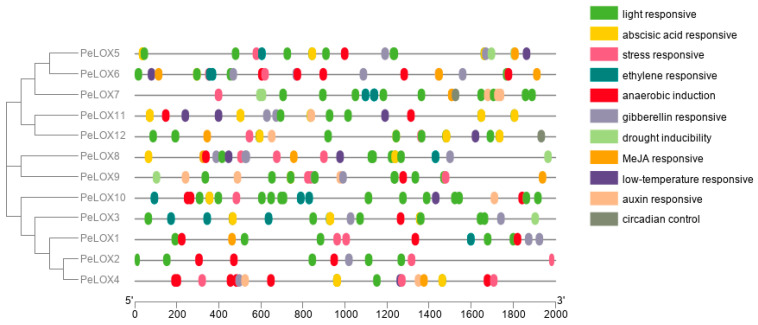
Cis-elements that are related to different stress and hormone in the putative promoters of *PeLOX*s.

Cis-acting elements are the binding sites of transcription factors and are involved in regulating the exact initiation and efficiency of gene transcription. Cis-acting element prediction could help us to further understand gene expression and regulation. Here, the −2000 bp sequence upstream of the *PeLOX* genes’ transcription start site was extracted and cis-acting elements were predicted using the PlantCARE online database. The prediction results showed that, in addition to the enhancer TATA box, CAAT box, and other elements, it also included light response elements G-box (TACGTG, CACGTG, CACGTC, CACGTT) and TCT-motif (TCTTAC), ABA response element ABRE (ACGTG), anaerobic sensing element ARE (AAACCA), gibberellin response element P-box (CCTTTTG), MeJA response element CGTCA-motif, and auxin response element TGA-element (AACGAC), stress response element STRE (AGGGG), ethylene-responsive element ERE (ATTTTAAA, ATTTCATA), and low-temperature response element LTR (CCGAAA) (Figure 5), indicating that the *PeLOX*s are involved in plant growth and development, hormone responses, and abiotic stress.

### 2.5. Expression Profile Analysis of PeLOX Genes

#### 2.5.1. Transcriptome Analysis under Different Abiotic Stresses

**Figure 6:**
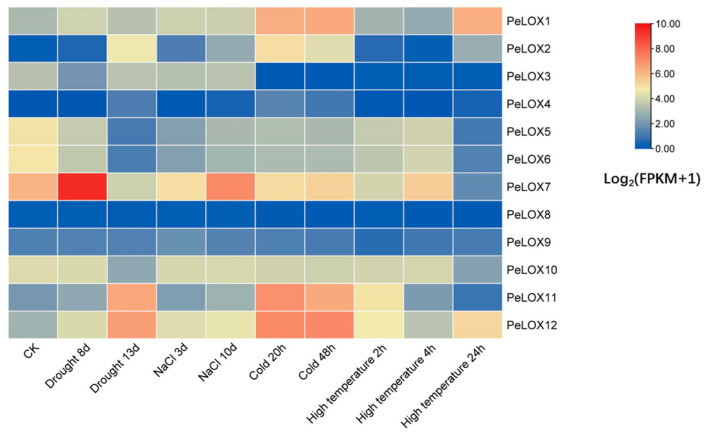
Heatmap of relative expression of *PeLOX* genes responding to drought, salt, cold, and high temperature via RNA-Seq. Blue indicates a low expression level and red indicates a high expression level.

RNA-seq data were used to study the expression profiles of *PeLOX* genes under different abiotic stresses (drought stress, salt stress, cold stress, and high-temperature stress) (Figure 6; Table S2). The results showed that the different *PeLOX* genes responded to different abiotic stresses to varying degrees, among which *PeLOX1*, *PeLOX2*, *PeLOX7*, *PeLOX11*, and *PeLOX12* responded significantly to various abiotic stresses, while *PeLOX8* and *PeLOX9* showed little change in expression under all stresses. The genes induced by drought stress included *PeLOX2*, *PeLOX4*, *PeLOX11*, and *PeLOX12*; the genes that first rose and then fell included *PeLOX1* and *PeLOX7*, among which *PeLOX7* had the highest expression level when the soil moisture was 50%; *PeLOX5*, *PeLOX6*, and *PeLOX10* were downregulated by drought stress; *PeLOX3* was first downregulated and then upregulated, while *PeLOX8* and *PeLOX9* were not induced by drought stress. Some genes were induced with the increase in salt stress, including *PeLOX1*, *PeLOX2*, *PeLOX11*, and *PeLOX12*; meanwhile, *PeLOX5*, *PeLOX6*, and *PeLOX7* showed first a downward and then an upward trend, among which *PeLOX7* had the highest expression level at 10 days of salt-stress treatment. The expression levels of *PeLOX3*, *PeLOX4*, and *PeLOX9* did not change significantly with the increase in salt stress. 

Under low-temperature stress, the expression of the *PeLOX1* and *PeLOX12* genes was upregulated, and the expression level of *PeLOX12* was the highest at 48 h of low-temperature treatment; *PeLOX3*, *PeLOX5*, and *PeLOX6* genes were downregulated by the low temperature. It is noteworthy that the expression levels of these upregulated and downregulated genes under a low temperature did not change significantly between different low-temperature treatment times. The expression levels of *PeLOX2* and *PeLOX11* first increased and then decreased; the *PeLOX7* expression level first decreased and then increased. In addition, there was no significant change in the expression of *PeLOX4*, *PeLOX8*, *PeLOX9*, and *PeLOX10*. Under high-temperature stress, the expression of most genes showed dynamic changes due to stress. The expression levels of *PeLOX1* and *PeLOX2* changed little between controls and those subjected to 4 h of high-temperature stress, but increased rapidly at 24 h of high-temperature stress; the *PeLOX3*, *PeLOX5*, *PeLOX6*, and *PeLOX10* expression levels were downregulated; the *PeLOX11* expression level was first upregulated and then downregulated; the *PeLOX7* expression level showed a dynamic change with a down–up–down trend, while *PeLOX12* showed a dynamic change with an up–down–up trend. In addition, the expression levels of *PeLOX4*, *PeLOX8*, and *PeLOX9* did not change significantly under high-temperature stress.

#### 2.5.2. Differential Expression in Seven Tissues

**Figure 7:**
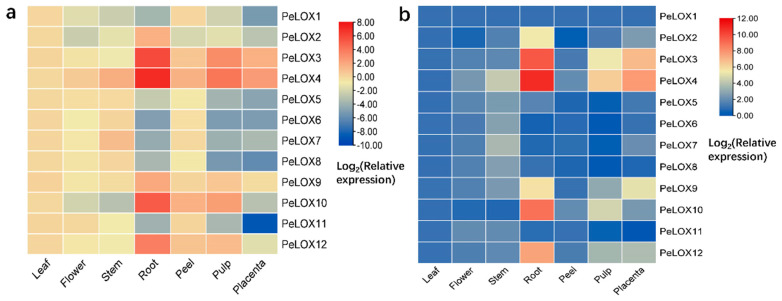
Expression profiles of *PeLOX* genes in various tissues of passion fruit. The expression of different *PeLOX* genes were normalized against the expression of leaf (**a**) and *PeLOX1* (**b**) in particular tissue. Blue indicates a low expression level and red indicates a high expression level.

To study the expression profiles of *PeLOX* genes in different tissues, seven different tissues of passion fruit, including leaves, flowers, stems, roots, pericarp, pulp, and placentation, were selected for qRT-PCR expression analysis (Figure 7; Table S3). In the standardization of the relative expression data, the expression of leaves was used as a reference. The results showed that *PeLOX* genes had obvious tissue expression specificity. Most of the genes are relatively more highly expressed in the roots, stems, and fruits, indicating that *PeLOX* genes mainly play a role in these tissues, while being relatively less present in other tissues. Among them, *PeLOX2*, *PeLOX3*, *PeLOX4*, *PeLOX9*, *PeLOX10*, and *PeLOX12* were highly expressed in the roots; *PeLOX4* and *PeLOX7* were highly expressed in the stems; *PeLOX3*, *PeLOX4*, and *PeLOX10* were highly expressed in the pulp, indicating that these genes may play a role in the pulp. In addition, *PeLOX3* and *PeLOX4* were relatively highly expressed in the placentas. It is noteworthy that *PeLOX4* was highly expressed in many tissues; thus, it may be a key lipoxygenase gene in passion fruit.

#### 2.5.3. Transcriptome Analysis (qRT-PCR) at Three Different Fruit Development Stages

**Figure 8:**
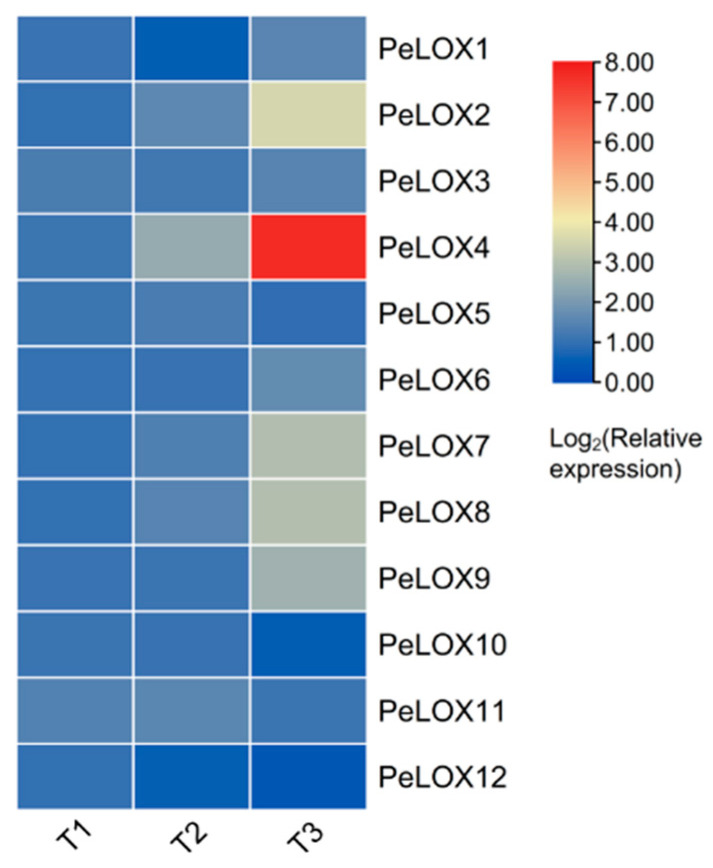
Relative expression of *PeLOX* genes at different fruit development stages from passion fruit via qRT-PCR.

The relative expression of *PeLOX* genes at three different fruit development stages in passion fruit was analyzed via qRT-PCR. The expression level of T1 was used as a reference in the standardization of the relative expression level data. The results (Figure 8; Table S4) showed that *PeLOX10* and *PeLOX12* decreased with the increase in fruit development and maturity, while *PeLOX2*, *PeLOX4*, *PeLOX6*, *PeLOX7*, *PeLOX8*, and *PeLOX9* were upregulated, and the qRT-PCR expression trend of *PeLOX2*, *PeLOX4*, *PeLOX10*, *PeLOX11*, and *PeLOX12* were consistent with the RNA-seq results (Figure S1). It is noteworthy that the expression level of *PeLOX4* was much higher than that of other family members, which was extremely significant, and the relative expression level of *PeLOX4* increased by 3.7 times and 46.5 times from T1 to T2 and from T2 to T3 with the increase in fruit development and ripeness, respectively. In addition, the expression level of *PeLOX3*, *PeLOX5*, and *PeLOX11* did not change significantly with fruit development and ripening.

### 2.6. Correlation Analysis of PeLOX4 Gene Expression, Enzyme Activity, and Fruit Development

**Figure 9:**
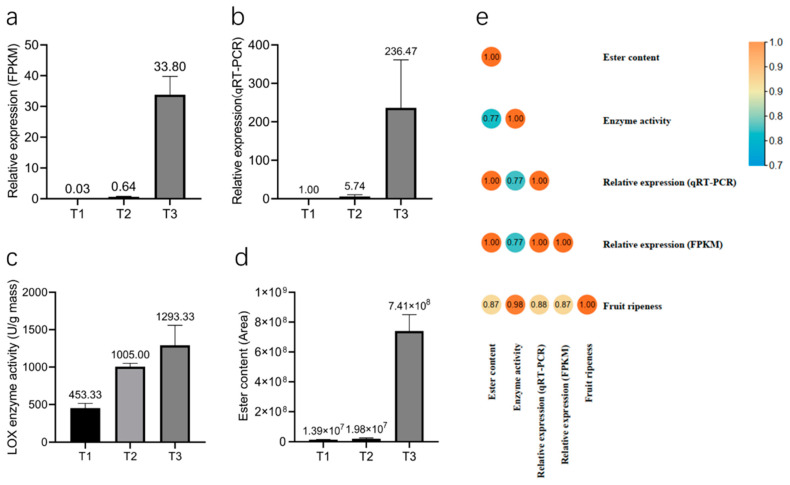
Histogram of *PeLOX4* relative expression, LOX enzyme activity and ester content in three different fruit development stages. (**a**) Histogram of *PeLOX4* relative expression via RNA-seq. (**b**) Histogram of *PeLOX4* relative expression via qRT-PCR. (**c**) Histogram of LOX enzyme activity. (**d**) Histogram of ester content. (**e**) Pearson correlation analysis of fruit ripeness, relative expression of *PeLOX4*, LOX enzyme activity and ester content.

The relative expression data of *PeLOX4* via the RNA-seq (Figure 9a) and the qRT-PCR (Figure 9b) results showed consistency, and *PeLOX4* was upregulated in response to the process of fruit development and ripening. During the ripening process of passion fruit, esters, especially those produced by the LOX pathway of fatty acid metabolism, play an important role in the composition of the fruit aroma. LOX was the first speed-limiting enzyme in the process of ester aroma synthesis. The LOX activity measurement results for three different fruit development periods (T1, T2, T3) are shown in Figure 9c. LOX enzyme activity was low in the T1 period, and rose rapidly in the T2 period. Until the T3 period, the enzyme activity was still in the rising stage, but the rate of rise slowed down. The content of esters (peak area) was extracted from the metabolome data of passion fruit at three different development stages (T1, T2, and T3), as shown in Figure 9d. With the development and ripening of the fruit, the ester content changed, increasing by 37.4 times from T2 to T3.

Pearson correlation analysis was conducted on fruit development and maturity, *PeLOX4* expression levels, LOX enzyme activity, and ester volatile substance content during the three different stages. The results are shown in Figure 9e (Table S5). The correlation coefficients reached 0.768 and above, indicating a high correlation. Among them, the correlation coefficient between *PeLOX4*′s relative expression and ester content was 1, indicating a complete correlation, and the correlation coefficient between fruit maturity and LOX enzyme activity was 0.984, which was extremely significant. In conclusion, the expression level of *PeLOX4*, LOX enzyme activity, and ester substance content were consistent with the increasing trend of fruit development and ripeness, and they had a correlation. It was concluded that with the increase in fruit ripeness, the expression level of *PeLOX4* increased, and LOX enzyme activity increased accordingly, thereby promoting the synthesis of volatile esters in fruit pulp.

## 3. Discussion

Lipoxygenase plays an important role in plant growth, development, stress resistance, maturation, and senescence, and has been widely studied in many species in recent years. In this study, 12 candidate *PeLOX* genes were identified by HMM and BlastP based on the genome annotation of passion fruit, and they were distributed on six chromosomes, among which *PeLOX2/3/4* and *PeLOX5/6/7* were tandem-linked and distributed on chromosomes 3 and 5, respectively. In cucumber, there were 23 *LOX* genes, and tandem duplication and/or polyploid duplication were the reasons for *CsLOXs’* amplification [16]. In poplar, the gene strings *PtLOX1/2/3/4*, *PtLOX 7/8/9/10*, and *PtLOX 16/17* formed by tandem repeat events were also found [25]. Gene duplication caused by tandem repeat events plays a vital role in the evolution of gene families. It could produce new genes and subfunctions or new functionalization, sharing the functions of ancestral genes in the manner of functional complementarity. Phylogenetic analysis showed that seven *PeLOX* proteins with the MdLOX1, MdLOX3, and MdLOX7 clusters belonged to the 9-LOX subfamily, including *PeLOX1/2/3/4/8/9/10*, and five *PeLOX* proteins with the MdLOX2, MdLOX5, MdLOX6, and MdLOX8 clusters belonged to the 13-LOX subfamily, including *PeLOX5/6/7/11/12* [7,18]. In gene structure analysis, it was found that all *PeLOX* proteins contained the highly conserved lipoxygenase domain, and some contained the relatively conserved PLAT domain. Among them, the lipoxygenase domain contained a conserved sequence of 38 amino acid residues, His-(X)_4_-HIS-(X)_4_-HIS-(X)_17_-HIS-(X)_8_-HIS, which was considered to be an important motif for the stability and activity of the LOX enzyme, and also provided a binding site for non-heme iron-containing dioxygenase [8]. This motif plays a significant role in binding iron atoms and maintaining the stability of the enzyme, and replacing any of the residues in the motif changes the activity of the LOX enzyme [25]. Similar results reported with the conserved motif were also obtained in the LOXs of melon [17], poplar [25], turnip [26], allotetraploid rapeseed [27], and tomato [20].

LOX generates oxylipins through enzymatic or non-enzymatic pathways, and the regulation of LOX enzyme activity and high transcriptional expression of the *LOX* gene are essential parts of oxylipins synthesis and stress response regulation [28]. Studies have shown that the expression of LOX is affected by many factors. For example, the expression of *PtLOX* in poplar was regulated by stress and methyl jasmonate (MeJA) [25]. The expression of melon *CmLOX09* was induced by auxin (indoleacetic acid (IAA)) and gibberellin (GA_3_) [29]. The reduction in the aroma of “Nanguoli” pear after cold storage was mainly regulated by fatty acid metabolic pathways, including the downregulated expression of *LOX2S*, *LOX1-5*, and other genes [30]. At the same time, MeJA could upregulate the expression of *AAT*, *ADH3*, *LOX1*, and other genes after cold storage, and the aroma compounds also increased [31]. Low-temperature storage also reduced the content of fruity note volatile esters and lactones in peach, and SA treatment could increase their volatile content; q-PCR analysis results showed that the transcription level of *PpLOX1* in peach fruit was significantly increased after SA treatment [32]. In this study, the cis-acting elements in the *PeLOX* genes’ promoter region were predicted, and results showed that the cis-acting elements included the MeJA response element, low-temperature response element, GA_3_ response element, stress response element, and auxin response element. The predicted results were consistent with the research results obtained in other species, suggesting that the expression of *PeLOX* genes may be regulated by the above predictive factors. 

In this study, qRT-PCR analysis of the leaf, flower, stem, fruit, root, and seed of passion fruit showed that *PeLOX* genes had obvious tissue expression specificity, and the expression levels of each *PeLOX* gene were different in various tissues. For example, the relative expression levels were higher in the roots, stems, and fruits, but lower in other tissues, indicating that the *LOX* genes may play a role in the high-expression tissues of passion fruit. Lipoxygenase of passion fruit in response to herbivory, insect, pathogen attack, and the application of jasmonate has been studied [9,10,11,12]. It was possible that the expression of the *LOX* gene promoted the synthesis of specific substances by participating in some important metabolic pathways. The tissue expression specificity of *LOX* has also been found in many other species. *AtLOX1* was found to be involved in the defense response in Arabidopsis leaves [33]; *AtLOX5* was highly expressed in the roots and was confirmed to affect the development of the lateral roots [34]. In persimmons, *DkLOX1* was specifically expressed in fruits, especially in young fruits, and *DkLOX3* was mainly expressed in mature fruits, especially during fruit storage; the overexpression of *DkLOX3* could accelerate fruit ripening and softening, while *DkLOX4* could be expressed in all tissues [35]. *PgLOX* genes were highly expressed in aerial parts, such as 3-year-old flowers, stems, and leaves [36].

The results of expression profile analysis confirmed that *PeLOX* genes were induced by abiotic stresses, including drought, salt, low-temperature, and high-temperature stress, among which *PeLOX1*, *PeLOX2*, *PeLOX7*, *PeLOX11*, and *PeLOX12* responded significantly to these abiotic stresses. It is speculated that the expression of the *PeLOX* genes increased the resistance of passion fruit to abiotic stress. A similar conclusion has been confirmed in many plants. Cucumber *LOX* transcripts showed differential accumulation or downregulation under plant growth regulators (MeJA, ACC, and ABA) and abiotic stresses (injury, low temperature, NaCl, and KCl) [24]. The expression of tomato *TomloxD* could be stimulated by wounding, pathogen infection, jasmonate, and systemin, and participated in the production of endogenous jasmonic acid, thereby regulating the expression of plant defense genes and resisting high-temperature stress and pathogen attack [37]. In pepper, *CaLOX1* plays an important role in the abiotic stress response by rapidly clearing ROS and activating defense-related marker genes [38]. Persimmon *DkLOX3* overexpression in Arabidopsis improved the tolerance to salt and drought stress [35]. Ginseng *13-LOX* positively responded to wounding and may have been involved in the production of C6 volatiles and jasmonic acid at the wounded sites, and the expression of *PgLOX3* was high under the conditions of water deficit, suggesting drought resistance in this gene [36]. Most cotton *GhLOX* genes were induced by heat and salt stress, and VIGS silencing of *GhLOX12* and *GhLOX13* led to a reduction in plant tolerance to salt stress, and confirmed the salt tolerance of both genes [39]. Tomato *LOX* genes selectively and differentially responded to heat, cold, drought, and salt stress [40]. Foxtail millet *SiLOX7* showed upregulated expression in two stress-tolerant varieties, indicating that it may play an important role in the response to salt and drought stress [41]. Subfamily members with different biological functions have also been studied. The number of 9-LOX and 13-LOX subfamilies varies in different plants, which may be related to differences in plant adaptation to the environment, e.g., the aquatic duckweed 13-LOX subfamily was associated with the synthesis of JA/MeJA, and its predominance in the Spirodela genome raises the possibility of a higher requirement for the hormone in the aquatic plant [42]. In the study of the interaction between sorghum and aphids, authors speculated that two 13-LOXs (SbLOX9 and SbLOX5) and three 9-LOXs (SbLOX1, SbLOX3, and SbLOXo) were involved in the biosynthesis of jasmonic acid, green leaf volatiles, and death acids, and all of them were involved in defense-related functions in plants [43]. Among the 12 *PeLOX* members, the 9-LOX and 13-LOX subfamily members totaled seven and five, respectively. *PeLOX4* belongs to the 9-LOX subfamily, and its high expression level was positively correlated with enzyme activity and ester content. Whether it was positively correlated with the increase in the content of jasmonic acid, volatiles, and death acids, thereby promoting the ability of passion fruit to resist pests and diseases, was a meaningful research question studied in depth. These studies were of great significance for the successful development of the passion fruit industry.

The expression specificity of *LOXs* in different tissues and stresses indicated that they played roles in regulating the growth and development of plant organs, enhancing the resistance of plants to biological and abiotic stresses, and producing plant-specific aromas. Among them, the regulation of LOX in the synthesis of specific volatile compounds during fruit development and maturation has been one of the research hotspots in recent years [44]. There are several pathways involved in volatile biosynthesis, starting from lipids, amino acids, terpenoids, and carotenoids, and the diversity of volatile compounds in fruits is achieved through the basic skeleton produced in these ways through additional modification reactions [44,45,46]. Different species of plants display different means of producing aromatic compounds, and most act through the LOX pathway, using fatty acids as the main precursors [6]. Transcriptome and metabolome analyses showed that *LOXs* could lead to differences in the strength of the “grassy” aroma between two white-fleshed pitaya cultivars [47]. Studies showed that the LOX enzyme activity in apple [48,49,50,51], strawberry [52,53], peach [28,54], pear [55], and grapes [56] was directly related to fruit ripening and fruit aroma formation. *TomLOXC* was confirmed to be involved in the production of fatty-acid-derived C5 and C6 flavor compounds [57,58]. *CmLOX03*, *CmLOX05*, *CmLOX11*, *CmLOX12*, *CmLOX16*, and *CmLOX18* are the key genes that affect aroma compounds, especially aldehydes and straight esters in melon [59], and *CmLOX18* could increase the synthesis of C6 volatiles through the HPL pathway [19]. In our previous study, results showed that with the increase in fruit ripeness, the total peak area of volatile aroma compounds increased by multiple times, and esters, especially the types and content of straight-chain esters, were the main components of the volatile aroma of passion fruit. Moreover, “α-linolenic acid metabolism” and the “secondary metabolism pathway” are the main pathways for the synthesis of important volatile aroma compounds in passion fruit; several enzyme genes of the fatty acid metabolism pathway, including *LOX*, *HPL*, *ADH*, and *AAT*, are expressed in response to the formation of esters [2]. In this study, the expression profiles of *PeLOX* genes in different fruit ripening stages were analyzed, among which the expression of *PeLOX4* showed a clear increase, increasing by 52.8 times from T2 to T3. Pearson correlation analysis was conducted on fruit development and maturity, *PeLOX4* expression levels, LOX enzyme activity, and ester volatile substance content at three different fruit stages. Results showed that the relative expression of *PeLOX4*, LOX enzyme activity, and ester content were highly consistent with the increasing trend of fruit development and maturity. It was inferred that the increase in *LOX* expression was related to fruit ripeness and ester synthesis. With the increase in fruit maturity, *PeLOX4* expression increased rapidly, LOX enzyme activity increased, and the synthesis of volatile esters in the pulp was promoted.

## 4. Materials and Methods

### 4.1. Identification of LOX Gene Family Members in Passion Fruit

Local BlastP (NCBI- Blast-2.11.0+), HMM searches and the *Passiflora edulis* genome data released by our research team (https://ngdc.cncb.ac.cn/gwh/Genome/557/show) were used for the identification of passion fruit LOX gene family members. Local BlastP were performed using the *AtLOX1*-*AtLOX6* full-length protein sequence from the *Arabidopsis thaliana* genome data (TAIR, http://www.Arabidopsis.org/) as the reference sequence. HMM searches were performed locally in the *P. edulis* protein database using the HMM profile of the ‘‘Lipoxygenase’’ domain PF00305 in Pfam (http://pfam.xfam.org/) with E-value = 10^−5^. In addition, all these obtained protein sequences were further analyzed by conservative domain filtering by the online program SMART (http://smart.embl/Heidelberg.de/). In total, 12 protein sequences were identified for further analysis. For phylogenetic analysis, the MdLOXs and SlLOXs were obtained using the same method in the *Malus domestica* (GDR, https://www.rosaceae.org/) [60] and *Solanum lycopersicum* (ITAG4.1) genome database.

Online tools EXPASY (https://web.expasy.org/protparam/) was used for protein physical and chemical parameters computation, WoLF PSORT (https://www.genscript.com/wolf-psort.html) was used for subcellular localization prediction, and NPSA (https://prabi.ibcp.fr/htm/site/web/home) for secondary structure prediction. TBtools software [61] was used for visualization of *PeLOX*s chromosome localization.

### 4.2. Phylogenetic, Gene Structure and Conserved Motifs Analysis

A multiple alignment analysis between the passion fruit, *Arabidopsis thaliana*, apple (*Malus domestica*), and tomato (*Solanum lycopersicum*) LOX proteins were carried out using MAFFT-7.490 (https://mafft.cbrc.jp/alignment/software/), and the Neighbor-Joining phylogenetic tree of these LOX proteins was created in MEGA X with bootstrap 1000. ChiPlot (https://www.chiplot.online/) was used to decorate beautification the phylogenetic tree.

Gene structural motif annotation was performed using the MEME (Multiple EM for Motif Elicitation, http://meme-suite.org/) program. Batch CD-Search domain (https://www.ncbi.nlm.nih.gov/Structure/bwrpsb/bwrpsb.cgi) was used for conservative structure prediction. The combined visualization of the phylogenic tree, the motifs, the conserved domain, and the gene structures of the *PeLOX* family were also visualized by TBtools software.

### 4.3. Cis-Acting Elements Prediction

The 2000 bp genomic DNA sequence upstream of the transcriptional start site in each *PeLOX* genes was filtered and uploaded to the online tool PlantCARE (http://bioinformatics.psb.ugent.be/webtools/plantcare/html/) database to identify the cis-acting elements in the promoter region of *PeLOX* genes.

### 4.4. Expression Profile Analysis and Plant Growth Conditions

To study the expression of *PeLOX* genes in different tissues, seven tissue sites of *Passiflora edulis* transplanted to the field for 4 months were selected, including leaf, flower, stem, root, peel, pulp, and placenta. Trizol (Invitrogen) was used for total RNA extraction, and Thermo Fermentas K1622 reverse transcription kit was used for cDNA synthesis. Fluorescence quantitative PCR was performed using Takara TB Green^®^ Premix Ex Taq™ II fluorescence quantitative PCR kit and Rothe Light Cycler96 real-time fluorescence quantitative PCR. The extension factor EF-1α was used as the reference gene, The extension factor EF-1α was used as an internal reference gene, and the primer sequences are shown in Table S6. Reaction conditions: 95 °C for 10 s, 60 °C for 10 s; 72 °C for 10 s, 45 cycles. After quantitative PCR, the relative expression of *PeLOX* genes were calculated by 2^−ΔΔCt^ method.

Healthy and virus-free passion fruit seedlings of purple fruit varieties were chosen for the expression analysis of *PeLOX* genes, which were grown in soil under a growth chamber (30 °C; 200 µmol·m^−2^·s^−1^ light intensity; 12 h light/12 h dark cycle; 70% relative humidity) to a height of about 1 m and with 8–10 functional leaves. For drought stress analysis, water was withheld from the treatment group to obtain soil moisture of 50% (8 days after stopping watering) and 10% (13 days after stopping watering), for salt stress tolerance analysis, the seedlings were treated with 300 mM NaCl solution after 3 and 10 days. For the cold treatment, the plants were treated at 0 °C for 20 h and 48 h, respectively. For the high temperature treatment, the plants were treated at 45 °C for 2 h, 4 h, and 24 h [62]. The germplasm of experimental materials related to fruit development was located in the passion fruit planting-base of CATAS, at 19 degrees north latitude, and the annual average temperature of the planting base was about 30 °C. In three different fruit maturity stages (T1, 2 weeks before harvest, the size of the fruit has been fully developed, and the peel is green; T2, at harvest time, the peel turns red without shrinkage; T3, 1 week after harvest at 30 °C, the peel has shrunk) [2] were extracted from the previously published RNA-seq data, and the expression heat map was analyzed using TBtools software.

### 4.5. qRT-PCR Verification

To verify the expression of *PeLOX* genes in different fruit maturity stages, we carried out the qRT-PCR verification using the same fruit samples as the RNA-seq. The genes with the same expression trend as RNA-seq and with significantly upregulated expression were selected to analyze the correlation between gene expression and fruit ripeness.

### 4.6. Correlation Analysis of PeLOX4 Gene Expression, Enzyme Activity and Fruit Ripeness

The LOX activity of fruit at three different fruit maturity stages were determined according to the procedures of the Plant Lipoxygenase (LOX) activity Detection Kit (Solarbio, Beijing, China). Previously published metabolome data were used for the statistics of volatile substance content of esters [2], calculated according to the total peak area of esters. SPSS 26 was used to analyze the Pearson correlation between fruit ripeness, *PeLOX4* expression (FPKM, qRT-PCR), LOX enzyme activity, and ester volatile substance content in three different fruit maturity stages.

## 5. Conclusions

In this study, we identified 12 lipoxygenase members from the passion fruit genome and carried out sequence analysis, phylogenetic analysis, gene structure analysis, and cis-acting element analysis. The expression profiles showed that *PeLOX* genes had tissue-specific expression. Some members responded to abiotic stresses, including drought, salt, low-temperature, and high-temperature stress, and participated in the process of fruit development and ripeness. We focused on *PeLOX4*, and the RNA-seq and qRT-PCR results showed that the relative expression of this gene increased rapidly with fruit development and ripeness. It is speculated that *PeLOX4* may be a candidate gene involved in fruit maturation and the formation of volatile aroma compounds. The results of enzyme activity determination and metabolome analysis showed that LOX enzyme activity increased significantly, and the content of esters increased with the development and ripeness of fruits, among which the ester content increased by 37.4 times from the T2 to T3 stage. Pearson correlation analysis confirmed the correlation between them, so it was inferred that the expression of *PeLOX4* increased, which in turn increased the LOX enzyme activity with the increase in fruit maturity, thus promoting the synthesis of volatile esters in the fruit pulp. The results of this study can improve the understanding of the function of the LOX gene in passion fruit, and contribute to the improvement of passion fruit quality and variety breeding.

## Figures and Tables

**Table 1:** The characteristics of 12 *PeLOX* members in *Passiflora edulis*.

Gene ID	Gene Location	CDS Length (bp)	Protein Length (aa)	Molecular Formula	Theoretical pI	Subcellular Location	Secondary Structure		
							Alpha Helix/%	Extended Strand/%	Random Coil/%
*PeLOX1*	P_edulis010002705.g	2688	895	C_4581_H_7094_N_1230_O_1356_S_24_	5.42	Cytoplasm	33.10	5.05	61.85
*PeLOX2*	P_edulis030007751.g	1587	528	C_2743_H_4284_N_730_O_792_S_14_	5.98	Cytoplasm	46.40	1.70	51.89
*PeLOX3*	P_edulis030007753.g	2610	869	C_4400_H_6816_N_1174_O_1321_S_20_	5.51	Nucleus	37.77	3.27	62.72
*PeLOX4*	P_edulis030007765.g	2610	869	C_4503_H_6977_N_1187_O_1314_S_19_	5.55	Cytoplasm	30.61	6.67	62.72
*PeLOX5*	P_edulis050012485.g	2697	898	C_4631_H_7151_N_1239_O_1341_S_21_	5.9	Chloroplast	32.63	5.26	62.11
*PeLOX6*	P_edulis050012486.g	2802	933	C_4822_H_7451_N_1297_O_1384_S_22_	6.27	Chloroplast	30.12	6.54	63.34
*PeLOX7*	P_edulis050012489.g	2679	892	C_4597_H_7088_N_1218_O_1321_S_19_	6.26	Chloroplast	33.97	4.37	61.66
*PeLOX8*	P_edulis060014414.g	2649	882	C_4500_H_7048_N_1270_O_1301_S_26_	9.16	Nucleus	31.63	8.39	59.98
*PeLOX9*	P_edulis060014719.g	1419	472	C_2443_H_3770_N_660_O_694_S_12_	6.5	Chloroplast	43.64	1.69	54.66
*PeLOX10*	P_edulis060016092.g	2601	866	C_4481_H_6950_N_1208_O_1300_S_28_	5.97	Cytoplasm	30.72	4.97	64.32
*PeLOX11*	P_edulis070018494.g	2643	880	C_4463_H_6996_N_1242_O_1308_S_22_	7.16	Chloroplast	33.89	5.73	60.38
*PeLOX12*	P_edulis090020726.g	2649	882	C_4475_H_7021_N_1233_O_1305_S_21_	6.93	Cytoplasm	37.59	4.01	58.40

## Data Availability

Not applicable.

## Supplementary Materials

The following supporting information can be downloaded at: https://www.mdpi.com/article/10.3390/ijms232012496/s1.
