# Supplementary figures and images for: Genome-Wide Association and Expression Analysis of the Lipoxygenase Gene Family in Passiflora edulis Revealing PeLOX4 Might Be Involved in Fruit Ripeness and Ester Formation
Source: Int J Mol Sci. 2022 Oct 18;23(20):12496. doi: 10.3390/ijms232012496 (PMC9603862; doi:10.3390/ijms232012496)

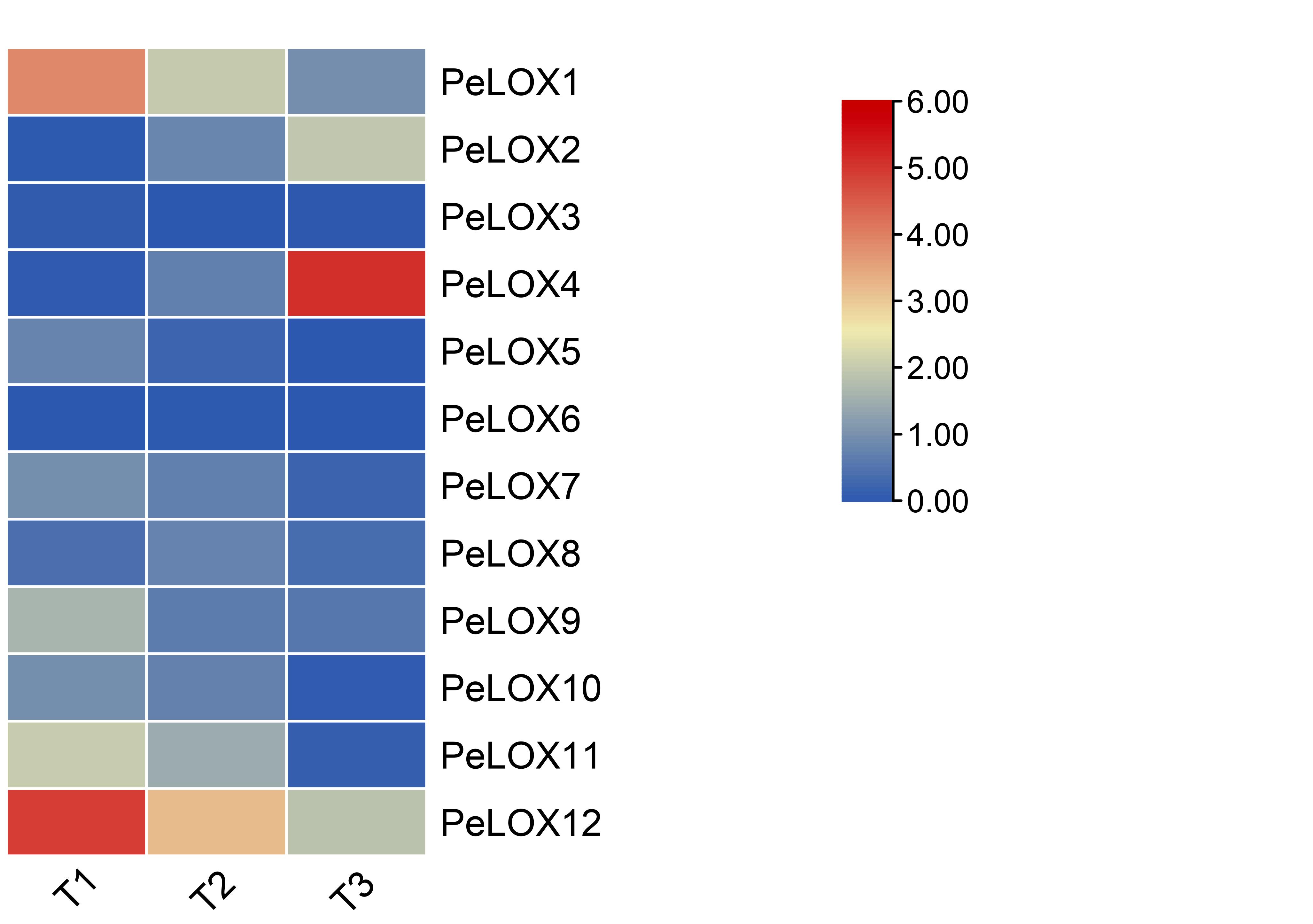

Supplement: Supplementary file 1 [file ijms-23-12496-s001.zip › ijms-1906030-supplementary/Additional fileú▌Figure S1.jpg]
